# Stimulus intensity and temporal configuration interact during bimodal learning and memory in honey bees

**DOI:** 10.1371/journal.pone.0309129

**Published:** 2024-10-03

**Authors:** Oswaldo Gil-Guevara, Andre J. Riveros

**Affiliations:** 1 Departamento de Biología, Facultad de Ciencias Naturales, Universidad del Rosario, Bogotá, Colombia; 2 Department of Neuroscience, College of Science, University of Arizona, Tucson, AZ, United States of America; University College London, UNITED KINGDOM OF GREAT BRITAIN AND NORTHERN IRELAND

## Abstract

Multimodal integration is a core neural process with a keen relevance during ecological tasks requiring learning and memory, such as foraging. The benefits of learning multimodal signals imply solving whether the components come from a single event. This challenge presumably depends on the timing and intensity of the stimuli. Here, we used simultaneous and alternate presentations of olfactory and visual stimuli, at low and high intensities, to understand how temporal and intensity variations affect the learning of a bimodal stimulus and its components. We relied on the conditioning of the proboscis extension response (PER) to train honey bees to an appetitive learning task with bimodal stimuli precisely controlled. We trained bees to stimuli with different synchronicity and intensity levels. We found that synchronicity, order of presentation, and intensity significantly impacted the probability of exhibiting conditioned PER responses and the latency of the conditioned responses. At low intensities, synchronous bimodal inputs produced maximal multisensory enhancement, while asynchronous temporal orders led to lower performances. At high intensities, the relative advantage of the synchronous stimulation diminished, and asynchronous stimuli produced similar performances. Memory retention was higher for the olfactory component and bimodal stimuli compared to the visual component, irrespective of the training’s temporal configuration. Bees retained the asynchronous bimodal configuration to a lesser extent than the synchronous one, depending on the stimulus intensity. We conclude that time (synchrony), order of presentation, and intensity have interdependent effects on bee learning and memory performance. This suggests caution when assessing the independent effects of each factor.

## Introduction

Multisensory integration is a phenomenon commonly observed across animal taxa and studied at several levels. At the processing level, specialized neural circuits discern whether sensory signals originate from the same event while computing the physical properties of the stimuli (e.g., intensity) [1–4]. Not surprisingly, multimodal integration is more robust when the signal components originate from the same location (following the so-called spatial rule) [3, 5, 6] and during temporal proximity (following the so-called temporal rule) [1, 6–9]. Nevertheless, neural integration should account for environmental variations in signal transmission. For instance, unimodal cues may be integrated into a single event within an ‘temporal window’ providing flexibility in handling temporal lags [10–14].

At both the neural and behavioural levels, multisensory integration, across ecological contexts, has long been associated with enhanced performance, spanning from invertebrates to humans [4, 7, 10, 15–22]. Such enhancement may derive, for instance, from higher information content within a signal and/or facilitated processing and integration [1, 10, 23]. Importantly, these benefits may contribute to explain the investments in multimodal signalling, such as increased signal conspicuousness and redundancy for senders, and enhanced signal detection and recognition for receivers [24–26]. Nevertheless, despite these advantages of multimodal signalling, exceptions exist that demonstrate variability in signalling effectiveness. For instance, bumble bees conditioned to a bimodal stimulus of blue light and 1-hexanol exhibited improved performance relative to bees conditioned to either the blue light or the 1-hexanol (the unimodal components) [27]. In contrast, bees trained to a bimodal stimulus of green light and linalool exhibited a higher performance than bees trained only with the green light (the unimodal visual component), but a lower performance relative to bees trained with linalool [27].

These observations opened the question of whether the properties of the conditioned bimodal stimuli, specifically the synchrony of presentation of the components and their intensity, may better explain the observed variations in performance. We addressed related questions in two previous studies. First, we showed that the intensity of components within a signal may impact performance following the so-called ‘Principle of Inverse Effectiveness’ (PoIE); [28]. According to the PoIE, combining low-intensity signals from multiple modalities should yield computational enhancements reflected at the neural and behavioural levels [1–3, 6, 23, 29]. While this may appear counterintuitive, it emphasizes the importance of relying on multiple sources of information when individual intensities may not suffice to fully solve a task (e.g., under dim light intensity or a weak odour plume). Second, we have shown that synchrony and identity of components within a cue may positively or negatively impact performance [27]. Specifically, presenting a visual component before the olfactory component enhanced performance and learning of the olfactory component. In contrast, the opposite structure (first olfactory and then visual) impaired learning of the overall cue and the visual component. This suggests a ‘supportive’ role of the visual component for the bees to learn the apparently preferred olfactory component [27]. Together, these results highlight the impact of the structure of the stimulus (independent of the components) and the intensity/identity of the components (independent of the structure) on both the acquisition and memory retention in bees. This opens several questions regarding the role of the structure within the signal and the identity of components at low intensities.

Here, we ask whether these previously observed effects of synchrony and identity of components (visual, olfactory) within a stimulus also interact with the intensity of the components. We were particularly interested in such interaction given our previous indications of the PoIE [28]. In nature factors such as light intensity, wind direction, and individual speed may simultaneously influence the saliency, sequence, and overlap of visual and olfactory stimuli [19, 20, 26, 27, 31–38]. For instance, in dense canopies, reduced light diminishes flower visibility, while wind alters odour plumes, influencing how pollinators perceive the timing and order of these stimuli. Thus, we aimed to integrate the main strengths of our previous findings [27, 28] to reach a more complete understanding of how bees integrate multimodal signals.

We relied on the bimodal conditioning of the proboscis extension response (PER) [30–36]. The PER is an innate feeding behaviour where bees extend their proboscis in response to sweet stimuli detected by their antennae or tarsi, mimicking nectar detection [31, 32]. In nature, the bees learn to associate some of the features of the flowers (e.g., colour, scent, shape) with the nectar. In an experimental context, such as classical conditioning of the PER, bees can be trained to associate a stimulus (e.g., an odour or colour: the conditioned stimulus, CS) [33] with a sucrose reward (the unconditioned stimulus, US). Eventually, a successful conditioning is indicated by a PER following the presentation of the CS alone (a conditioned PER, cPER), implying the acquisition of the association [30]. This method has been widely utilized to study cognitive functions in bees [36]. Honey bees typically exhibit higher acquisition and retention with olfactory versus visual stimuli in the PER conditioning protocol [35, 37, 38]. However, using Africanized honey bees rather than European honey bees has enabled successful colour conditioning using the PER protocol [33], allowing the study of multimodal conditioning [28]. While this protocol has a constrained scope compared to free flight experiments, it enables *i*. precise control of stimulus delivery, *ii*. precise variation of intensity, *iii*. accurate quantification of latency of response, *vi*. the ability to work with larger sample sizes.

Thus, we specifically evaluated the effect of conditioning with bimodal stimuli (visual: V; olfactory: O) with different temporal configurations (synchrony, asynchrony) and, in the asynchronous stimuli, at alternate temporal orders (OV or VO). Each temporal configuration was presented at high and low intensities. We predicted that honey bees would exhibit higher levels of conditioned PER for synchronous bimodal stimuli compared to asynchronous stimuli (rendering support for the temporal rule, see above). Our design also allowed us to investigate the potential interaction between intensity and synchrony. We predicted a reduced difference in the PER response of bees between synchronous and asynchronous stimuli at high intensities. Moreover, following our previous findings [27, 28], we also studied the impact of the temporal order of the unimodal elements during asynchronous stimulation. Hence, we analysed whether stimulus order affects bees’ conditioned response. We conducted memory retention tests to examine the persistence of effects observed during acquisition after 24 hours. The test aimed to determine whether bees learned each element of both synchronous and asynchronous conditions independently of the temporal configuration, and whether learning was influenced by the sequence in which different modalities were presented (olfactory, then visual and vice versa) or based on temporal proximity to the reward (unconditioned stimuli). Finally, we aimed to identify differentiated recall between synchronicity and intensity levels during the memory test, revealing crossmodal interactions caused by temporality and intensity.

## Materials and methods

### Animal handling

We employed Africanized honey bees (*Apis mellifera*), from the apiary of the Universidad Nacional de Colombia (Bogotá; ≈2600m elevation). We collected worker bees leaving the hive (13:00h-16:00), using an acrylic pyramid [30]; all bees were from the same colony. We ice anaesthetized [33] and harnessed the bees into custom-made 3D-printed plastic tubes [28]. The harnessing procedure involved placing the anesthetized bee in the tube with its legs resting inside. A thin string of adhesive paper tape was used to hold the back of the head, only allowing the movement of the proboscis. A piece of non-adhesive tape immobilized the wings and back of the bee. A few minutes after recovery, we fed the bees to satiation with sucrose-water (1.5M) and kept them under laboratory conditions (natural photoperiod, RH: 58%). The following morning (~10 min before the onset of the experiments), we tested bees’ motivation using the innate PER elicited by the antennal stimulation with sucrose water. We solely included motivated individuals in the experiments. After training and memory tests, we marked surviving bees on the thorax using Testors® paint, a reliable labelling method for honey bees [39, 40]. Subsequently, we released them near the laboratory, approximately 1 km from the hives, ensuring a single experimentation *per* individual bee.

### Automatized training device

**Fig 1:**
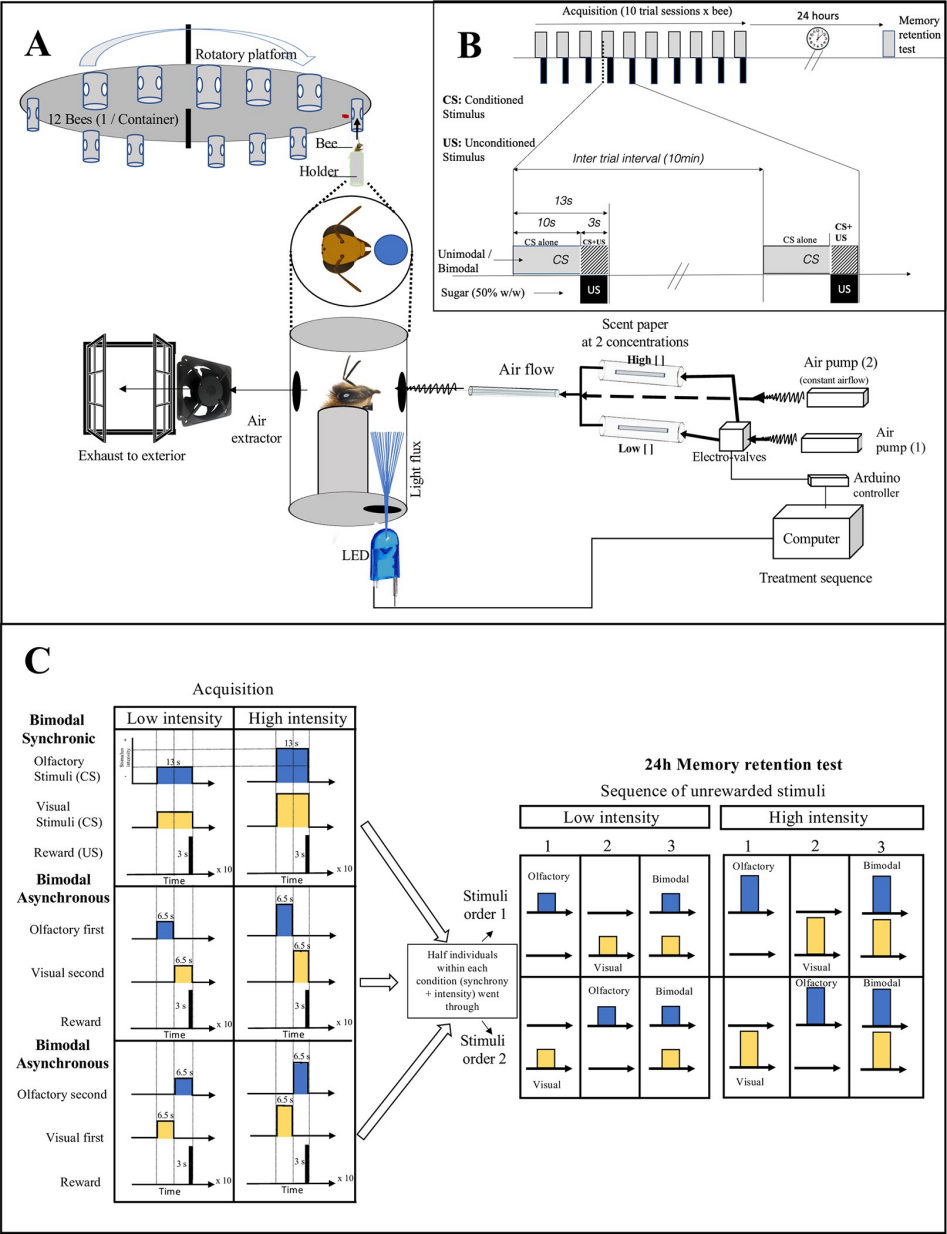
Experimental device and protocol. (A) Training apparatus. The system controlled olfactory and visual stimuli with varying intensities. (B) Conditioning protocol. (C) Overall experimental design. Left: synchronous and asynchronous stimuli presented at low or high intensity. Right: Memory retention tests for unimodal components (note balanced order of presentation) and bimodal synchronous configuration.

We employed a rotary training device [27, 33, 41, 42] modified to allow automatic and precise delivery of olfactory and visual stimuli [28]. Briefly, the rotary setup (diam.: 0.52 m) contained 12 chambers, each one covered with aluminium foil tape on the inside to homogenize the light reflectance from a LED positioned below the chamber (the variation inside the chamber was not measured; Fig 1A). Every chamber had two openings: at the front and the back, allowing the flow of pumped air through the enclosure and access for the experimenter to manually deliver the sucrose reward. The training device was connected to a computer-controlled system to deliver visual and olfactory stimuli at different intensities and temporal orders. The stimuli followed a pre-programmed sequence, [28], controlled by a PC running Processing software (v. 3.5.3) [43] manually initiated by the experimenter (see full details on software control in [28].

A set of parallel electronic valves allowed airflow from a pump into one of two parallel glass tubes holding filter paper with a scent molecule at a particular concentration. We prepared olfactory stimulation by soaking a piece of filter paper (~10x4mm) with 10 μL with a molecule in solution at the corresponding concentration (low/high, Fig 1; see below). Filter paper was replaced after three consecutive training trials using the same odour concentration (low or high). This ensured consistent olfactory stimulation, as preliminary calibrations indicated the paper became dryer after three training trials of use. The airflow (flow rate: 1.08 L/min; Fluke VT Plus HF gas flow analyser) reached the test chambers after mixing it with a parallel and constant flow of unscented air (flow rate: 0.33 L/min) aimed to minimize the possible effect of mechanical stimulation (Fig 1A). The constant flow also cleaned the residual odour traces between trials. The tubing system and glassware was made of laboratory-grade materials.

Light intensity was controlled by automatically varying the electric current. The software code implemented also allows registering the time of behavioural events (latency of a conditioned PER). The position of the harnessed bees inside the apparatus and the ubication of the LED light source (see Fig 1A) might have allowed a direct light illumination only at the lower portion of the bee’s eyes.

### Variation of stimuli intensity

We varied the intensity of the visual and olfactory stimuli following our previous measures of near-threshold parameters [28]. Briefly, the near low threshold level was determined as the minimum magnitude leading to a conditioned response that differed from a negative control and that simultaneously differed from the learning performance achieved with higher intensities. Visual stimulation was supplied by a blue LED (peak λ = 458 nm) that shone from below inside the chamber (Fig 1A) within a range of intensities from 0 to 45.7 μmol photons m^-2^s^-1^ (LI-COR portable spectroradiometer, model Li-1400, Lincoln, NE, USA). Low intensity was 10% of the maximum LED emission (4.6 μmol photons m^-2^s^-1^), and high intensity was 100% of maximum emission (45.7 μmol photons m^-2^s^-1^). For olfactory stimulation, we used 1-Hexanol 98% (Sigma-Aldrich #H13303). We diluted or not the scent in mineral oil to achieve a high (7.8 M; 98%) or a low concentration (2x10^-4^ M; 0.025%). For bimodal stimulation, we programmed the device to provide visual and olfactory stimuli according to the experimental design.

### Experimental design

We aimed to assess the impact of two factors: (i) stimulus intensity and (ii) the bimodal signal structure, including the synchrony between components and the order of presentation. For this, we randomly assigned each bee to one of six treatments: synchronous bimodal (Sync: visual + olfactory: O+V), or asynchronous (olfactory and then visual: OV; or, visual and then olfactory: VO; Fig 1B and 1C). Each treatment was delivered at either low or high intensity of its components. During each training session, two bees representing each treatment were randomly assigned to chambers within the setup.

### Training and testing procedure

We trained honey bees using an absolute conditioning learning task, modifying the original PER protocol [30–32, 36] as described by Jernigan (2014) and Gil-Guevara et al. (2022). Prior to each training trial, bees were acclimated for 15 seconds. During training, the conditioned stimulus was presented for 13s. After 10s of presentation, the experimenter approached a micropipette with a drop of sucrose water (1.5M), stimulated the antenna (which elicited a PER) and allowed the bee to drink during the last 3s of exposure to the conditioned stimulus. The training trial ended with 15s of defamiliarization before a new bee was positioned for training. When a bee exhibited a conditioned PER (cPER), we directly allowed the bee to drink for 3s.

During the asynchronous training, bees received the first component during the initial 6.5 seconds, followed by the second component during the last 6.5 seconds (Fig 1C). Each bee underwent 10 training trials with an average 10-minute intertrial interval (Fig 1B). During conditioning and memory trials, we recorded the latency of cPER. After eliciting a PER, the experimenter pressed a key on the computer keyboard, and the custom software displayed the elapsed time from stimulus presentation to PER onset on the screen. After 24h, we evaluated memory retention by presenting sequentially either the visual or olfactory components and then the bimodal stimulus at the intensity used during training (Fig 1C). This form of retention test allowed us to identify whether the bees learned a single component, both, or the configuration and how this was impacted by the conditions used during training [27]. We balanced the presentation of the sequence by randomly assigning 50% of bees to each group (Fig 1C). With this we aimed to balance the potential effect of extinction induced by the unrewarded presentation of the components. Furthermore, we presented the bimodal stimulus after the unimodal stimuli to reduce potential extinction effects from the stronger, unrewarded bimodal stimulus.

### Statistical analyses

*Learning performance* was calculated based on the binary cPER responses during each training trial, where bees could exhibit or not a cPER. We assessed the potential effects of exposure to the six treatments and performance changes across training trials by constructing a Generalized Linear Mixed Model with a bimodal link function. We assigned the structure of the signal (synchrony level/modality order: 3 levels: Sync, VO, OV), the intensity (2 levels: high, low), the interaction between them (structure*intensity), and the training trial (the within-subject factors / repeated measures) as independent variables. We included the individual as a random factor. Similarly, during the memory retention test we relied on a GLMM model as explained above but excluded the training trial as an independent variable. Instead, we added *Modality* as a factor, aiming to test the differences in performance depending on the modality used during training (olfactory, visual, bimodal).

*Latency of conditioned responses* during acquisition, was calculated as the average latency for individuals exhibiting at least three cPERs (N = 253 out of 300). We employed a Generalized Linear Mixed Model (GLMM) with a Gamma distribution and log link for latency analysis, appropriate for the positively skewed and strictly positive data [44–49]. For acquisition, the model included Structure (modality order), Intensity, and Trial as independent variables, with a secondary analysis examining variations within Structure, across intensity levels (low vs high). During the memory retention test, we assessed responses to unrewarded unimodal (olfactory, visual) and bimodal stimuli in bees previously trained under different stimulus Structures.

We executed the analyses in R v.4.0.3. (http://www.R-project.org/) employing the package “lme4” [44]. This package allows carrying GLMMs with either a binomial error distribution or a Gamma distribution and log link using the function glmer(). We selected the most appropriate model based on the lowest Akaike Information criterion score (AIC) from various combinations of independent variables and interactions, without applying a specific AIC difference threshold, ensuring an optimal balance of simplicity and explanatory power [45, 50]. We used χ^2^ analysis for GLMMs with the "Anova" function from the car() package [48, 49] to test individual factors’ effects [45, 48, 51]. We further used the package emmeans() to conduct pairwise comparisons (Tukey HSD method with Bonferroni correction), estimated marginal means (EMMS), odds ratios, and predicted probabilities [52].

## Results

We captured and harnessed 486 worker bees. Only bees exhibiting a PER to antennal stimulation with the sucrose solution were included in experiments. Thus, we excluded 186 bees and distributed 300 bees across the six treatments.

### Acquisition

**Fig 2:**
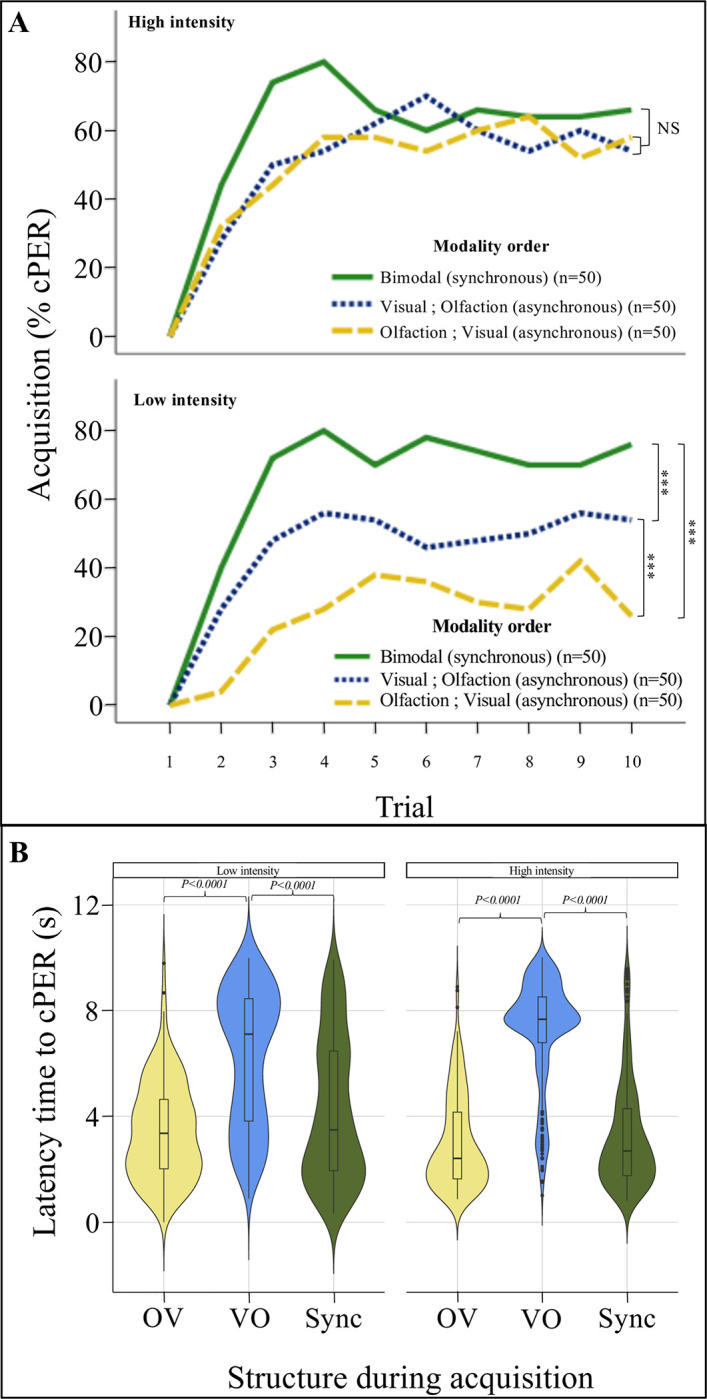
Performance during acquisition and latency of response. (A) Performance of bees across training trials when trained at high (upper panel) or low intensity (lower panel). (B) Latency of conditioned responses during the training phase.

Bees in all treatments exhibited increased probabilities of cPERs throughout training trials, supporting the effectiveness of the training protocol (GLMM: Trial: χ^2^_1,300_ = 33.17; *P*<0.0001; Fig 2A; S1A Table). The probability of a bee exhibiting a cPER was significantly impacted by the structure of the bimodal signal (GLMM: Structure: χ^2^_2,300_ = 38.94; *P*<0.0001; S1A Table), and the intensity of the stimuli (GLMM: Intensity: χ^2^_1,300_ = 7.73; *P* = 0.005; S1A Table).

Moreover, the effect of these factors on the performance was significantly impacted by their interaction (GLMM: Structure x Intensity: χ^2^_2,300_ = 15.69; *P*<0.001; Fig 2A; S1A Table) such that the effect of the structure differed depending on the intensity. At low intensities, the performance of the bees trained with the synchronous stimulus was significantly higher than the performance of the bees trained to either asynchronous stimuli (Sync vs. OV: Tukey: Z = 7.14; odds ratio = 8.2; estimate = -2.10; *P<*0.0001; Sync vs. VO: Tukey: Z = 3.72; odds ratio = 2.93; estimate = -1.07; *P* = 0.0006; Fig 2A). Moreover, within the asynchronous stimuli, bees in the VO group exhibited significantly higher performances than bees in the OV group (OV vs. VO, Tukey: Z = -3.56; odds ratio = 2.8; estimate = -1.03, *P* = 0.001). In contrast, at high intensity, the performance of the bees did not significantly differ between bees trained with different structures of the stimuli (Sync vs. OV: Tukey: Z = 1.71; odds ratio = 1.63; estimate = -0.49; *P* = 0.20; Sync vs. VO: Tukey: Z = 1.57; odds ratio = 1.57; estimate = -0.45; *P* = 0.26; OV vs. VO, Tukey: Z = -0.14; odds ratio = 1.04; estimate = -0.04; *P* = 0.99; Fig 2A).

Remarkably, intensity also appeared to have a heterogeneous effect within treatments. Bees trained with the OV stimulus exhibited a significantly higher performance when trained with high than with low intensities (GLMM, Low vs. High intensity: χ^2^_1,100_ = 19.75; *P*<0.0001; Table 1A). In contrast, intensity did not significantly affect the performance of bees trained with the VO (GLMM: Low vs. High intensity: χ^2^_1,100_ = 1.74; *P* = 0.19; Table 1B) or the Sync stimulus (GLMM: Low vs High intensity: χ^2^_1,100_ = 0.80; *P* = 0.37; Table 1C).

**Table 1:** Contrasts within stimulus structure across intensity levels. Summary of GLMM and LMM comparing the performance during acquisition (A, B, C) and the latency of cPER (D, E, F) between low and high intensities for each structure of the bimodal stimulus (OV, VO, and Sync).

**A)**	**GLMM for cPER of bees trained using the OV stimulus at low and high intensities**				
	= PER ~ OV (Low) + OV (High) + Trial + (1|Individual)				
	**Structure (Coefficients)**	**Estimate**	**SE**	**Z**	***P***
	(Intercept) OV (Low)	-1.815	0.269	-6.736	<0.0001
	OV (High)	1.369	0.308	4.444	<0.0001
	Trial	0.124	0.026	4.724	<0.0001
**B)**	**GLMM for cPER of bees trained using the VO stimulus at low and high intensities**				
	= PER ~ VO (Low) + VO (High) + Trial + (1|Individual)				
	(Intercept) VO (Low)	-0.506	0.216	-2.344	0.0191
	VO (High)	0.320	0.243	1.320	0.1868
	Trial	0.081	0.024	3.393	0.0007
**C)**	**GLMM for cPER of bees trained using the Sync stimulus at low and high intensities**				
	= PER ~ Sync (Low) + Sync (High) + Trial + (1|Individual)				
	(Intercept) Sync (Low)	0.751	0.270	2.786	0.0053
	Sync (High)	-0.286	0.321	-0.891	0.3727
	Trial	0.048	0.027	1.790	0.0735
**D**)	**LMM for latency of bees trained using the OV stimulus at low and high intensities**				
	= Latency (s) ~ OV (Low) + OV(High) + Trial + (1|Individual)				
	**Synchronicity level (Coefficients)**	**Estimate**	**SE**	**t**	***P***
	(Intercept) OV (Low)	0.14	0.08	1.86	0.06
	OV (High)	1.12	0.07	14.97	<0.001
	Trial	-0.008	0.01	-0.49	0.62
**E)**	**LMM for latency of bees trained using the VO stimulus at low and high intensities**				
	= Latency (s) ~ VO (Low) + VO (High) + Trial + (1|Individual)				
	(Intercept) VO (Low)	-0.15	0.04	-4.06	<0.001
	VO (High)	1.91	0.04	45.74	<0.001
	Trial	0.01	0.01	2.01	0.04
**F)**	**LMM for latency of bees trained using the Sync stimulus at low and high intensities**				
	= Latency ~ Sync (Low) + Sync (High) + Trial + (1|Individual)				
	(Intercept) Sync (Low)	0.24	0.07	3.40	<0.001
	Sync (High)	1.22	0.07	17.91	<0.001
	Trial	-0.01	0.031	-1.29	0.20

### Latency of conditioned responses during acquisition

**Fig 3:**
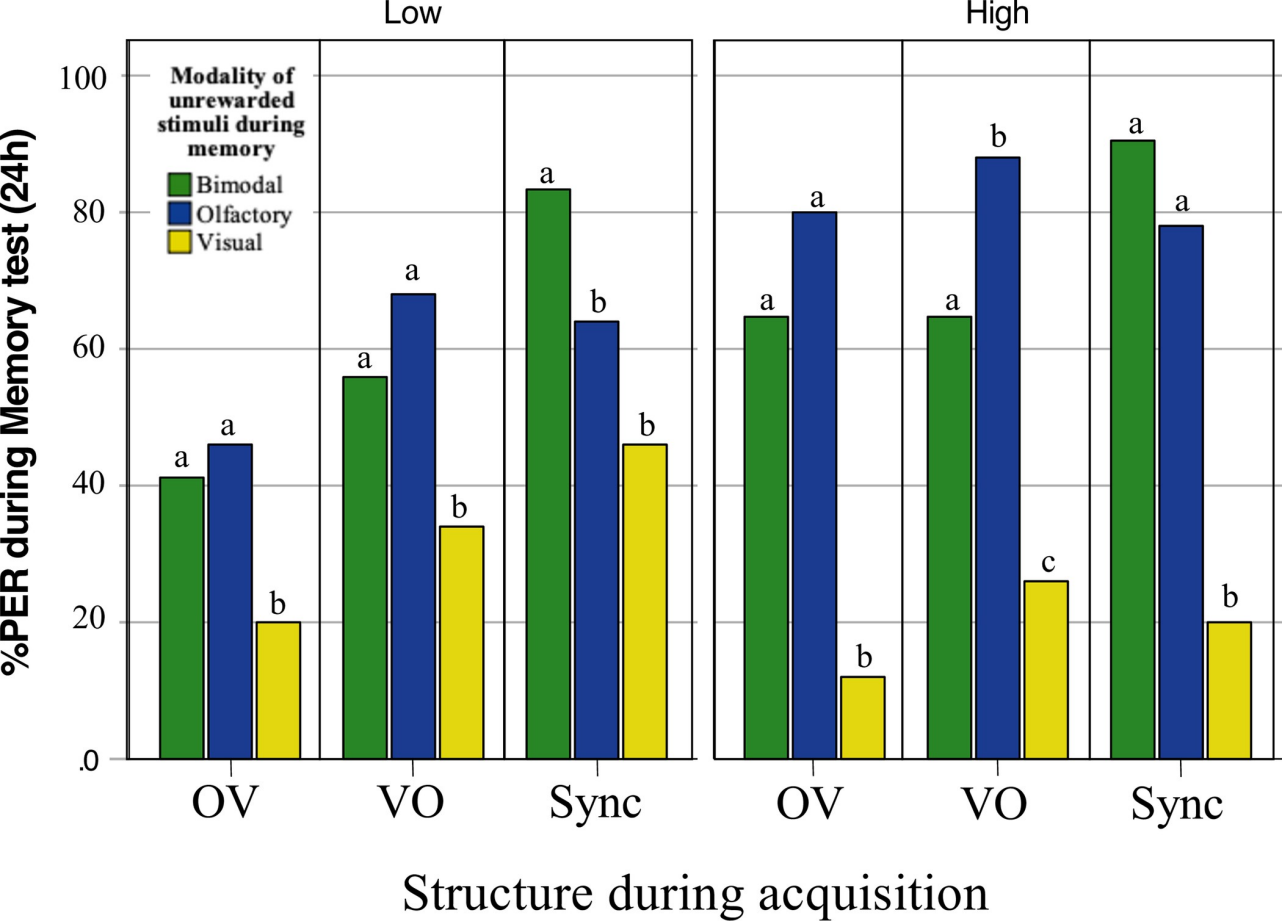
PER response during memory retention test. Conditioned responses to components during the retention test 24h after conditioning. P values obtained using the Tukey HSD method.

The latency to exhibit a cPER response was significantly affected by the structure of the stimuli (GLMM: Structure: χ^2^_2,274_ = 315.5; *P*<0.0001; S1B Table) but remained unchanged across trials within treatments (GLMM: Trial: χ^2^_1,274_ = 0.2145; *P* = 0.64; S1B Table). The stimulus intensity also affected the latency of cPER (GLMM: Intensity: χ^2^_1,274_ = 4.468; *P* = 0.035; S1B Table), and significantly interacted with the structure (LMM: Structure x Intensity: χ^2^_2,274_ = 21.83; *P*<0.0001; S1B Table). Bees trained with the VO stimulus exhibited significantly longer latencies of cPERs (i.e., slower responses) at low and high intensities than bees trained with the OV or the Sync stimuli (Low intensity: VO vs. OV: z = -8.105, *P*<0.0001; VO vs. Sync: z = 6.91, *P*<0.0001; High intensity: OV vs. VO: z = -14.33, *P*<0.0001; VO vs. Sync: z = 13.54, *P*<0.0001; Fig 2B). In contrast, we did not observe any significant differences between the latencies of bees trained with the Sync and the OV stimuli either at high or low intensities (Low intensity: OV vs. Sync: z = -2.32, *P* = 0.053; High intensity: z = -1.12; *P* = 0.5; Fig 3B).

Interestingly, the latency time of cPER was heterogeneously affected by intensity of stimuli. Bees trained with the OV stimulus did not exhibit latencies significantly different between intensities (Low vs. High intensity: χ^2^_1,83_ = 3.44; *P* = 0.06; Table 1D). In contrast, bees trained with the VO stimulus exhibited significantly longer latencies of cPER at high intensities (Low vs. High intensity: LMM χ^2^_1,95_ = 16.52; *p*<0.00001; Table 1E) and bees trained with the Sync stimulus exhibited significantly shorter latencies of cPER at high intensities (Low vs. High intensity effect: LMM χ^2^_1,96_ = 11.56; *P* = 0.001; Table 1F).

### Memory retention after 24h

We found that the probability of exhibiting a cPER after 24h was significantly associated with the structure of the stimulus used during training, the unrewarded stimulus presented during the retention test (O, V, Sync) and their interaction (GLMM: *Structure during acquisition*: χ^2^_2,300_ = 8.46; *P* = 0.02; *Modality tested*: χ^2^_4,300_ = 135.87; *P*<0.0001; *Structure during acquisition x Modality tested*: χ^2^_4,300_ = 15.350; *P* = 0.004; S2 Table). Interestingly, the intensity of stimuli during training did not significantly affect the probability of exhibiting a cPER during the memory test (GLMM: Intensity: χ^2^_1,300_ = 1.54; *P* = 0.22; S2 Table). Yet, we found a significant interaction between intensity of stimuli and modality tested (GLMM: *Intensity x Modality tested*: χ^2^_4,300_ = 23.92; *P*<0.0001; S2 Table).

We evaluated the cPER recall of bees trained with each stimulus structure (OV, VO, O+V) at low and high intensities during the acquisition phase and subsequently tested with unrewarded unimodal (V, O) and bimodal (O+V) stimuli. For bees trained with the OV structure, no significant differences were observed in the recall of olfactory and bimodal stimuli at either intensity (Post hoc, Tukey: Low intensity: Z = -0.22, estimate = -0.13, P = 0.97; High intensity: Z = -1.42, estimate = 0.62, P = 0.33; Fig 3, S3 Table). However, recall of the visual element was significantly lower than both the bimodal and olfactory stimuli across all intensities (Low intensity: B vs V: Tukey: Z = 2.61, estimate = 1.65, P = 0.03; High intensity: B vs V: Tukey: Z = 5.11, estimate = 3.65, P<0.0001; Low intensity: O vs V: Tukey: Z = 3.19; estimate = 1.78; P = 0.004; High intensity: O vs V: Tukey: Z = 6.41; estimate = 4.52; P<0.0001; Fig 3, S3 Table).

Bees trained with the VO structure at low intensities exhibited a similar recall pattern, with comparable recall for olfactory and bimodal stimuli (B vs O: Tukey: Z = -1.05, estimate = -0.60, P = 0.54; Fig 3, S3 Table), but significantly reduced recall for the visual element (B vs V: Tukey: Z = 2.56, estimate = 1.49, P = 0.03; O vs V: Tukey Z = 3.90, estimate = 2.10, P < 0.00001; Fig 3, S3 Table). At high intensities, olfactory elements were recalled better than bimodal stimuli (B vs O: Tukey: Z = -2.42, estimate = -1.71, P = 0.04; Fig 3, S3 Table), while the visual element continued to show the lowest recall levels (B vs V: Tukey: Z = 4.0, estimate = 2.57, P < 0.00001; O vs V: Tukey Z = 6.12, estimate = 4.28, P <0.0001; Fig 3, S3 Table).

Conversely, bees trained with synchronous bimodal stimuli at low intensity exhibited the highest recall for bimodal stimuli compared to unimodal elements (B vs O: Tukey: z = 2.36, estimate = -1.51, P = 0.05; B vs V: Tukey: z = 4.07, estimate = 2.65, P <0.00001; Fig 3, S3 Table), although at low intensity, olfactory and visual stimuli were recalled at similar levels, the differences were not statistically significant (O vs V: Tukey z = 2.21, estimate = 1.14, P = 0.07; Fig 3, S3 Table). At high intensity, performance on bimodal and olfactory stimuli was equivalent (B vs O: Tukey: z = 1.62, estimate = 1.13, P = 0.24; Fig 3, S3 Table), yet visual stimuli recall remained significantly lower (B vs V: Tukey: z = 6.11, estimate = 4.6, P < 0.0001; O vs V: Tukey z = 5.76, estimate = 3.48, P <0.001; Fig 3, S3 Table).

The latency of cPER by bees during the memory tests was influenced by the modality tested (GLMM: Modality tested: χ^2^_2, 238_ = 119.97; *P*<0.0001; S4A Table), and the intensity of the stimulus (GLMM: Intensity: χ^2^_1,238_ = 4.75; *P*<0.05) but not their interaction (GLMM: Modality tested X Intensity: χ^2^_2,238_ = 4.07; *P* = 0.13). In addition, the structure of the bimodal signal during acquisition, did not significantly affect the latency of cPERs during the memory test (GLMM: Structure: χ^2^_2,238_ = 1.41; *P* = 0.49; S4A Table). Bees consistently exhibited longer cPER latencies when stimulated with the visual component compared to both olfactory and bimodal stimuli, across all intensity levels (Low intensity: O vs V: Tukey z = -6.06, estimate = -0.55, P<0.0001; High intensity: O vs V: Tukey z = -7.04, estimate = -0.78, P<0.0001; Low intensity: B vs V: Tukey z = -5.72, estimate = -0.57, P<0.0001; High intensity: B vs V: Tukey z = -7.21, estimate = -0.85, P<0.0001; S4B Table). The cPER latencies for bees stimulated with olfactory and synchronous bimodal stimuli were similar at both low and high intensities (Low intensity: Tukey z = 0.22, estimate = 0.02, P = 0.97; High intensity: Tukey z = -0.98, estimate = -0.07, P = 0.59).

## Discussion

Multimodal integration facilitates the concurrent processing of information from different sensory modalities, playing a crucial role in decision-making across taxa [6, 53–57]. In pollinators such as bees, this ability should enable more efficient use of signals emitted by flowers [20, 58]. Despite the widely accepted prediction that multimodal signals lead to improved performance [19, 59–61], their effectiveness depends on specific physical properties of the stimuli, whose interactions—following earlier work [24, 25, 35, 57, 62–66] and our previous findings [27, 28, 67]—remain to be understood, especially in the context of learning and memory. Here, we expand this work by examining how timing and intensity interact in bees’ learning and memory, determining whether the structure of a bimodal signal—synchrony and order of presentation—and intensity impact performance. We found that the structure significantly affected performance only at low intensities. Bees trained with the synchronous stimulus exhibited the highest performance, followed by intermediate performance with the VO stimulus (first visual, then olfactory), and the lowest performance with the OV stimulus (first olfactory, then visual). The latency of the conditioned response varied with treatment and intensity. After 24 hours, during a memory test, the cPER was significantly impacted by the training conditions and the tested modality. The lowest response level was observed with visual stimulation, whereas the highest responses occurred with synchronous and olfactory stimulation. Our results can be explained by the interaction of three key elements: the temporal rule of integration, the Principle of Inverse Effectiveness, and sensory biases.

### Temporal rule of integration

First, honey bees trained and tested with the bimodal synchronous signal (Sync) consistently exhibited high levels of cPER and short latencies of response (i.e., fast responses). This pattern agrees with the so-called temporal rule of multimodal integration [4, 6, 9, 23]. Accordingly, optimal enhancement is expected when stimuli are presented approximately at the same time, as the brain perceives them as from the same event [9, 10, 23]. Interestingly, we previously found that bees performed best with a partially asynchronous stimulus (visual followed by olfactory) [27]. This configuration was not tested in the current study, limiting direct comparisons. Remarkably, here bees trained with a synchronous stimulus consistently showed high performance, with significant differences from asynchronous stimuli only at low intensities. This aspect was not explored in the previous study [27], which used only high-intensity stimuli. Thus, at low intensities, full synchrony may be more relevant for learning the overall stimulus (Fig 2A; see further discussion on intensity below), while at high intensities, asynchronous patterns may play a more significant role for learning a more relevant component (Fig 3). In support of this hypothesis, bees generally achieved similar conditioned response levels at high intensities. However, the strongest olfactory response was observed when bees were trained with an asynchronous stimulus, starting with a visual component followed by an olfactory component (Fig 3). This suggests that the relevance of synchrony in multimodal integration depends on stimulus intensity, with full synchrony being more effective at low intensities and asynchronous patterns more beneficial for learning specific components at high intensities.

The temporal rule underscores the importance of synchrony in learning and memory [1, 6–9, 68], while a related concept, the temporal window, explores the specific time range for effective multisensory integration [10–12, 14]. In our experiment, at high intensities, bees trained with asynchronous stimuli performed similarly to those with synchronous stimuli, indicating that temporal asynchrony did not break the temporal window. However, at low intensities, bees trained with synchronous stimuli maintained high performance, while those with asynchronous stimuli exhibited decreased performance, especially in the OV condition (Fig 2A; Table 1). This suggests that the effectiveness of the temporal window depends on stimulus intensity, in line with theoretical predictions [10, 11]. Overall, our findings support the temporal rule of integration and highlight the role of intensity in modulating the temporal window’s effectiveness. Further research is needed to explore the exact limits and mechanisms of this temporal window in bees during similar tasks.

Our findings have important ecological implications for foraging insects, particularly bees. Variations in temporal synchrony likely enhance pollinator efficiency by optimizing the use of floral signals at different stages of foraging. In natural environments, bees likely encounter the visual component of floral signals early during flower detection, with olfactory cues becoming more relevant as they approach or land on the flower [69–71]. Such a temporal sequence aligns with the temporal rule of multimodal integration, enhancing bees’ foraging efficiency as needed. Our data suggest a dynamic interaction between synchrony and intensity, which may be part of the adaptive strategies in sensory processing that evolved during the coevolution of pollinators and plants [20].

### Principle of Inverse Effectiveness (PoIE)

Second, while the spatial and temporal rules depict intrinsic stimulus properties, the PoIE describes an inverse relationship between unisensory responsiveness and the overall effectiveness of multisensory integration. It emphasizes that multisensory stimuli are more effectively integrated when individual unisensory responses are weak, predicting greater integration at the lowest stimulus intensities [2–6, 10, 23, 29, 72, 73]. Our current results align with our previous findings, indicating that the integration of olfactory and visual components is more significant at low intensities [28]. Importantly, the reduced performance in bees trained with asynchronous conditions suggests again, an interaction between the PoIE and the temporal rule of multimodal integration. Bees trained with synchronous stimuli performed significantly better than those in the asynchronous groups, indicating that their performance was not driven by a single sensory component. Rather, the bees integrated the signal from both modalities to improve their performance at the more challenging task. In turn, this indicates that the bees successfully learned the composed signal through training.

Interestingly, during the initial training trials, high-intensity synchronous stimuli enhanced learning more rapidly than asynchronous stimuli (Fig 2A). However, this advantage faded, resulting in similar performance across stimulus structures by the end of training (Sync vs. OV and VO). We interpret these transient boosts as likely reflecting attentional mechanisms rather than lasting cognitive processes [74–76]. Further analyses on stimuli synchronicity’s impact on learning speed across intensity conditions could clarify distinctions between attentional and acquisition processes.

Building on these insights, our acquisition and memory results (Figs 2 and 3) align with previous research showing that the temporal configuration can influence whether one sensory modality facilitates the acquisition of another component or the full configuration [27, 35, 63]. In addition, our study reveals a previously unreported interdependency between timing and intensity during the acquisition and memory of multisensory stimuli, underscoring the importance of considering both factors in multimodal research. Moreover, our findings align with previous research on crossmodal interactions, demonstrating that bees can recall specific colours alongside an odour scent during reward reinforcement and beyond [64, 77]. In our study, the VO combination enhances acquisition and memory recall more than the OV combination, consistent with findings from previous studies [27, 35]. Additionally, our results show that intensity levels influence the strength of these recollections (Fig 3), providing insights into how bees encode, store, and retrieve information. This ability may benefit foraging bees through cross-modal information transfer across varying intensities [78].

### Sensory biases

Lastly, we suggest that sensory biases impacted the performance of the bees across treatments. Bees exhibit an olfactory bias relative to visual information during conditioning [37, 42, 63, 66, 79–81]. This bias means that bees preferentially learn and respond to olfactory cues over visual ones, regardless of intensity and temporal order. Consequently, in both unimodal and multimodal training, olfactory cues have a stronger associative strength. The reduced learning in the OV condition may result from the temporal separation between the odour and the reward, as optimal learning occurs when these stimuli overlap by about 1 second [31, 36, 82]. Alternatively, the order of the modalities might modulate the acquisition of the preferred olfactory component, as reported in other studies [35, 83]. In our experiment, the VO condition had the reward more closely associated with the olfactory cue, enhancing learning and recall. Conversely, in the OV condition, the visual cue’s proximity to the reward did not significantly enhance learning, as bees inherently favour olfactory cues. Despite the asynchrony in OV leading bees to receive the reward during the visual component, they still preferentially responded to the odour during the test. When the olfactory component was preceded by the visual component (VO), bees increased their response to the olfactory cue, supporting the idea that the visual component aids olfactory learning [27]. Additionally, during memory retention, the latency of conditioned responses to visual stimulation was longer (see results; S4 Table), indicating slower processing times. Not surprisingly, the speed of conditioned responses increased with intensity in bees tested with the Olfactory and Bimodal stimuli.

Our findings indicate that bees exhibit a natural bias towards olfactory cues over visual ones during conditioning, leading to stronger and faster learning when olfactory stimuli are involved. Performance was driven by the integration of both modalities, indicating strong learning of the olfactory component and significantly lower learning of the visual component (Fig 2). The poor response rate to the visual stimulus during the memory test (around 20%) suggests that the visual component alone did not effectively support learning. Such low associative power of visual stimuli might result from the reported decline of bees’ visual performance at both low and high intensities attributed to the specific response function of the lamina monopolar cells [84–86]. Therefore, the significant differences in performance between the synchronous (B), VO, and OV conditions might underscore the critical role of olfactory stimuli in enhancing learning and memory recall in bees. Further research should explore these temporal dynamics, such as reversing the reward order while alternating olfactory and visual elements, to better understand the interplay between sensory modalities and temporal factors in bee learning.

## Conclusions

Our study highlights the critical interaction between temporal synchrony and stimulus intensity in bee multimodal learning and memory. At low intensities, synchrony significantly enhances conditioned responses compared to asynchronous configurations, but this advantage diminishes at high intensities. These results provide a more cohesive understanding of factors affecting performance in multimodal tasks. Our findings confirm the natural bias of bees towards olfactory cues over visual ones, resulting in stronger and faster learning with olfactory stimuli in both unimodal and multimodal training. Future research should explore a broader range of intensity levels and temporal configurations to better understand sensory integration dynamics and natural foraging conditions. Precise quantification of the temporal window’s range in multisensory integration should yield new insights into its effects on attention and learning in bees. To enhance the precision of PER latency measurements, future studies should consider utilizing video analysis or automated tracking systems. Additionally, examining the neural mechanisms underlying these processes will deepen our understanding of how bees integrate and process multimodal signals. Our contribution demonstrates the interaction between temporal structure and intensity during multimodal learning and memory. The observed interactions between synchrony, temporal order, and intensity reveal a previously unreported interdependency, which should be considered in future studies of multimodal learning. These interactions enhance foraging efficiency through cross-modal information transfer, a key adaptive strategy in the coevolution of pollinators and plants. Our findings provide a foundation for further research into the complexities of sensory integration in ecological contexts.

## Supporting information

S1 Table Generalized linear mixed model (GLMM) models during acquisition. **A)** GLMM (bimodal link function) model for the effect of stimulus Structure, Intensity and Trial on the probability of eliciting a cPER response during the acquisition phase of associative conditioning experiments on bees. **B)** GLMM (Gamma distribution and log link) model exploring the change in the latency time (s) as a function of the stimulus Structure, intensity level, and trials during acquisition. Significance levels are assessed after Bonferroni’s correction.
(DOCX)

S2 Table Summary of a binomial GLMM model the cPER response of honey bees during the three phases of the memory retention test (see methods). The model test three coefficients: Stimulus Structure received during acquisition (three levels), intensity level (two levels) and modality order received during the three-phase memory test (five levels). Model fit: link function (logit); marginal / conditional R2 = 0.25/0.69, AIC = 873.91, ICC = 0.59, individual honeybees denoted as random effects = 300. Follow up chi-square test shown in text (see methods, results). Significance values following a Bonferroni’s correction: **<0.01; *<0.05.
(DOCX)

S3 Table Contrasts for the memory retention tests. Post hoc contrasts following a GLMM model contrasting the effect of the stimulus Structure employed during acquisition, and the order of presentation on the PER of honey bees during the balanced memory test. Confidence level used: 0.95. Results given on the log odds ratio scale; p values obtained using the Tukey HSD method.
(DOCX)

S4 Table Effects on latency time during memory test. **A.** LMM model to test the effects of stimulus Structure during acquisition, Intensity and the modality order employed during memory tests on latency time. Significance values following a Bonferroni’s correction. **B.** Posthoc contrasts after the LMM model contrasting the difference in the latency time between modalities of unrewarded stimuli during the memory retention test.
(DOCX)
