# Supplementary material for: Stimulus intensity and temporal configuration interact during bimodal learning and memory in honey bees
Source: PLoS One. 2024 Oct 3;19(10):e0309129. doi: 10.1371/journal.pone.0309129 (PMC11449348; doi:10.1371/journal.pone.0309129)
Supplement: S1 Table — A) GLMM (bimodal link function) model for the effect of stimulus Structure, Intensity and Trial on the probability of eliciting a cPER response during the acquisition phase of associative conditioning experiments on bees. B) GLMM (Gamma distribution and log link) model exploring the change in the latency time (s) as a function of the stimulus Structure, intensity level, and trials during acquisition. Significance levels are assessed after Bonferroni’s correction. (DOCX) [file pone.0309129.s001.docx]

**Supporting information for:**

**Stimulus Intensity and Temporal Configuration Interact During Bimodal Learning and Memory in Honey Bees**

Oswaldo Gil-Guevara^1*^ and Andre J. Riveros^1,2,*^

^1^Departamento de Biología, Facultad de Ciencias Naturales, Universidad del Rosario. Cra. 26 #63B-48. Bogotá. Colombia

^2^ Department of Neuroscience, School of Brain, Mind and Behavior. University of Arizona, Tucson, AZ, 85721

^*^Authors for correspondence ([oswaldo.gil.g@urosario.edu.co](mailto:oswaldo.gil.g@urosario.edu.co) ; [ajosafat@arizona.edu](mailto:ajosafat@arizona.edu))

# Supporting information

**S1 Table. Generalized linear mixed model (GLMM)** **models during acquisition. a)** GLMM (bimodal link function) model for the effect of stimulus Structure, Intensity and Trial on the probability of eliciting a cPER response during the acquisition phase of associative conditioning experiments on bees. **b)** GLMM (Gamma distribution and log link) model exploring the change in the latency time (s) as a function of the stimulus Structure, intensity level, and trials during acquisition. Significance levels are assessed after Bonferroni’s correction.

| **a) GLMM Acquisition Model for PER** | | | | | |  | **b) GLMM Acquisition Model for Reaction time (s)** | | | | |
| --- | --- | --- | --- | --- | --- | --- | --- | --- | --- | --- | --- |
| cPER response ~ Structure *X* Intensity + Trial + (1 \| individual) | | | | | |  | Latency time ~ Modality Order *X* Intensity + Trial + (1 \| individual) | | | | |
|  |  |  |  |  |  |  |  |  |  |  |  |
|  |  |  |  |  |  |  | |  |  |  |  |
|  | Estimate | S.E. | z value | *P-value* |  | Estimate | | S.E. | t value | *P-value* |  |
| Intercept | -1.567 | 0.225 | -6.962 | **<0.0001** |  | 1.250 | | 0.060 | 19.740 | **<0.0001** |  |
| (Structure) Visual, then Olfactory | 1.030 | 0.289 | 3.559 | **<0.001** |  | 0.590 | | 0.070 | 8.110 | **<0.0001** |  |
| (Structure) Bimodal | 2.105 | 0.295 | 7.145 | **<0.0001** |  | 0.160 | | 0.070 | 2.320 | **0.002** |  |
| (Intensity) High | 1.330 | 0.290 | 4.584 | **<0.0001** |  | -0.140 | | 0.070 | -2.010 | 0.040 |  |
| Trial | 0.084 | 0.015 | 5.759 | **<0.0001** |  | -0.001 | | 0.001 | -0.460 | 0.640 |  |
| (Structure) Visual, then Olfactory X (Intensity) High | -0.989 | 0.405 | -2.440 | <0.05 |  | 0.300 | | 0.100 | 3.140 | **0.0020** |  |
| (Structure) Bimodal X (Intensity) High | -1.614 | 0.411 | -3.925 | **<0.0001** |  | -0.090 | | 0.090 | -1.000 | 0.3100 |  |
|  |  |  |  |  |  |  | |  |  |  |  |
|  |  |  |  |  |  |  | |  |  |  |  |
