# Supplementary material for: Stimulus intensity and temporal configuration interact during bimodal learning and memory in honey bees
Source: PLoS One. 2024 Oct 3;19(10):e0309129. doi: 10.1371/journal.pone.0309129 (PMC11449348; doi:10.1371/journal.pone.0309129)
Supplement: S2 Table — The model test three coefficients: Stimulus Structure received during acquisition (three levels), intensity level (two levels) and modality order received during the three-phase memory test (five levels). Model fit: link function (logit); marginal / conditional R2 = 0.25/0.69, AIC = 873.91, ICC = 0.59, individual honeybees denoted as random effects = 300. Follow up chi-square test shown in text (see methods, results). Significance values following a Bonferroni’s correction: **<0.01; *<0.05. (DOCX) [file pone.0309129.s002.docx]

**Supporting information for:**

**Stimulus Intensity and Temporal Configuration Interact During Bimodal Learning and Memory in Honey Bees**

Oswaldo Gil-Guevara^1*^ and Andre J. Riveros^1,2,*^

^1^Departamento de Biología, Facultad de Ciencias Naturales, Universidad del Rosario. Cra. 26 #63B-48. Bogotá. Colombia

^2^ Department of Neuroscience, School of Brain, Mind and Behavior. University of Arizona, Tucson, AZ, 85721

^*^Authors for correspondence ([oswaldo.gil.g@urosario.edu.co](mailto:oswaldo.gil.g@urosario.edu.co) ; [ajosafat@arizona.edu](mailto:ajosafat@arizona.edu))

# Supporting information

**S2 Table. Summary of a binomial GLMM model the cPER response of honey bees during the three phases of the memory retention test (see methods).** The model test three coefficients: Stimulus Structure received during acquisition (three levels), intensity level (two levels) and modality order received during the three-phase memory test (five levels). Model fit: link function (logit); marginal / conditional R2=0.25/0.69, AIC = 873.91, ICC = 0.59, individual honeybees denoted as random effects=300. Follow up chi-square test shown in text (see methods, results). Significance values following a Bonferroni’s correction: **<0.01; *<0.05.

| **GLMM Memory Model for PER** |  |  |  |  |  | |  |
| --- | --- | --- | --- | --- | --- | --- | --- |
| PER response ~ Structure during acquisition X Intensity X Unrewarded stimuli during memory test + (1 \| individual honeybee) | | | | | |  | |
|  | **Estimate** | **Std.error** | **z value** | ***p*** |  | |  |
| Intercept | -0.3097 | 0.5556 | -0.5570 | 0.5772 |  | |  |
| (Structure / acquisition) Visual; Olfactory | 0.8108 | 0.7767 | 1.0440 | 0.2966 |  | |  |
| (Structure / acquisition) Bimodal | 2.6293 | 0.8270 | 3.1790 | **0.0015** | ** | |  |
| (Intensity) High | 1.3263 | 0.7859 | 1.6880 | 0.0915 |  | |  |
| (Stimuli during memory) Olfactory | 0.1276 | 0.5810 | 0.2200 | 0.8262 |  | |  |
| (Stimuli during memory) Visual | -1.6526 | 0.6339 | -2.6070 | **0.0091** | ** | |  |
| (Structure / acquisition) Visual; Olfactory X (Intensity) High | -0.7647 | 1.1171 | -0.6850 | 0.4937 |  | |  |
| (Structure /acquisition) Bimodal X (Intensity) High | -0.8572 | 1.2023 | -0.7130 | 0.4759 |  | |  |
| (Structure /acquisition) Visual; Olfactory X (stimuli during memory) Olfactory | 0.4741 | 0.8153 | 0.5810 | 0.5609 |  | |  |
| (Structure /acquisition) Bimodal X (stimuli during memory) Olfactory | -1.6372 | 0.8636 | -1.8960 | 0.0580 |  | |  |
| (Structure /acquisition) Visual; Olfactory X (stimuli during memory) Visual | 0.1593 | 0.8613 | 0.1850 | 0.8533 |  | |  |
| (Structure /acquisition) Bimodal X (stimuli during memory) Visual | -0.9990 | 0.9087 | -1.0990 | 0.2716 |  | |  |
| (Intensity) High X (stimuli during memory) Olfactory | 0.7495 | 0.8473 | 0.8850 | 0.3764 |  | |  |
| (Intensity) High X (stimuli during memory) Visual | -1.9947 | 0.9542 | -2.0900 | 0.0366 | * | |  |
| (Structure / acquisition) Visual; Olfactory X (Intensity) High X (stimuli during memory) Olfactory | 0.3602 | 1.2428 | 0.2900 | 0.7720 |  | |  |
| (Structure / acquisition) Bimodal X (Intensity) High X (stimuli during memory) Olfactory | -0.3674 | 1.2700 | -0.2890 | 0.7724 |  | |  |
| (Structure /acquisition) Visual; Olfactory X (Intensity) High X (stimuli during memory) Visual | 0.9191 | 1.2895 | 0.7130 | 0.4760 |  | |  |
| (Structure /acquisition) Bimodal X (Intensity) High X (stimuli during memory) Visual | 0.0431 | 1.3792 | 0.0310 | 0.9751 |  | |  |
