# Supplementary material for: Stimulus intensity and temporal configuration interact during bimodal learning and memory in honey bees
Source: PLoS One. 2024 Oct 3;19(10):e0309129. doi: 10.1371/journal.pone.0309129 (PMC11449348; doi:10.1371/journal.pone.0309129)
Supplement: S3 Table — Post hoc contrasts following a GLMM model contrasting the effect of the stimulus Structure employed during acquisition, and the order of presentation on the PER of honey bees during the balanced memory test. Confidence level used: 0.95. Results given on the log odds ratio scale; p values obtained using the Tukey HSD method. (DOCX) [file pone.0309129.s003.docx]

**Supporting information for:**

**Stimulus Intensity and Temporal Configuration Interact During Bimodal Learning and Memory in Honey Bees**

Oswaldo Gil-Guevara^1*^ and Andre J. Riveros^1,2,*^

^1^Departamento de Biología, Facultad de Ciencias Naturales, Universidad del Rosario. Cra. 26 #63B-48. Bogotá. Colombia

^2^ Department of Neuroscience, School of Brain, Mind and Behavior. University of Arizona, Tucson, AZ, 85721

^*^Authors for correspondence ([oswaldo.gil.g@urosario.edu.co](mailto:oswaldo.gil.g@urosario.edu.co) ; [ajosafat@arizona.edu](mailto:ajosafat@arizona.edu))

# Supporting information

**S3 Table. Contrasts for the memory retention tests.** Post hoc contrasts following a GLMM model contrasting the effect of the stimulus Structure employed during acquisition, and the order of presentation on the PER of honey bees during the balanced memory test. Confidence level used: 0.95. Results given on the log odds ratio scale; p values obtained using the Tukey HSD method.

| **Contrasts between unrewarded stimulation during memory tests** | **Stimulus structure and intensity during acquisition:** | | | | | | | | | |  |
| --- | --- | --- | --- | --- | --- | --- | --- | --- | --- | --- | --- |
|  | **Low intensity** | | | |  |  | **High intensity** | | | |  |
|  | **Olfactory; Visual (O; V)** | | | |  |  | **Olfactory; Visual (O; V)** | | | |  |
|  | **estimate** | **SE** | **z. ratio** | ***p*** |  |  | **estimate** | **SE** | **z. ratio** | ***p*** |  |
| Bimodal - Olfactory | -0.128 | 0.581 | -0.220 | 0.974 |  |  | -0.877 | 0.617 | -1.422 | 0.329 |  |
| Bimodal - Visual | 1.653 | 0.634 | 2.607 | **0.025** | * |  | 3.647 | 0.713 | 5.114 | **<.0001** | *** |
| Olfactory - Visual | 1.780 | 0.558 | 3.190 | **0.004** | ** |  | 4.524 | 0.705 | 6.414 | **<.0001** | *** |
|  |  |  |  |  |  |  |  |  |  |  |  |
|  | **Visual; Olfactory (V; O)** | | | |  |  | **Visual; Olfactory (V; O)** | | | |  |
| Bimodal - Olfactory | -0.602 | 0.572 | -1.052 | 0.544 |  |  | -1.711 | 0.707 | -2.422 | **0.041** | * |
| Bimodal - Visual | 1.493 | 0.583 | 2.561 | **0.028** | * |  | 2.569 | 0.642 | 4.001 | **<.00001** | **** |
| Olfactory - Visual | 2.095 | 0.538 | 3.896 | **<.00001** | **** | | 4.280 | 0.700 | 6.117 | **<.0001** | *** |
|  |  |  |  |  |  |  |  |  |  |  |  |
|  | **Bimodal in-sync** | | | |  |  | **Bimodal in-sync** | | | |  |
| Bimodal - Olfactory | 1.510 | 0.639 | 2.362 | **0.048** | * |  | 1.127 | 0.698 | 1.616 | 0.239 |  |
| Bimodal - Visual | 2.652 | 0.651 | 4.073 | **<.00001** | **** | | 4.603 | 0.753 | 6.110 | **<.0001** | *** |
| Olfactory - Visual | 1.142 | 0.518 | 2.207 | 0.070 |  |  | 3.476 | 0.604 | 5.757 | **<.0001** | *** |
