# Supplementary material for: Stimulus intensity and temporal configuration interact during bimodal learning and memory in honey bees
Source: PLoS One. 2024 Oct 3;19(10):e0309129. doi: 10.1371/journal.pone.0309129 (PMC11449348; doi:10.1371/journal.pone.0309129)
Supplement: S4 Table — A. LMM model to test the effects of stimulus Structure during acquisition, Intensity and the modality order employed during memory tests on latency time. Significance values following a Bonferroni’s correction. B. Posthoc contrasts after the LMM model contrasting the difference in the latency time between modalities of unrewarded stimuli during the memory retention test. (DOCX) [file pone.0309129.s004.docx]

**Supporting information for:**

**Stimulus Intensity and Temporal Configuration Interact During Bimodal Learning and Memory in Honey Bees**

Oswaldo Gil-Guevara^1*^ and Andre J. Riveros^1,2,*^

^1^Departamento de Biología, Facultad de Ciencias Naturales, Universidad del Rosario. Cra. 26 #63B-48. Bogotá. Colombia

^2^ Department of Neuroscience, School of Brain, Mind and Behavior. University of Arizona, Tucson, AZ, 85721

^*^Authors for correspondence ([oswaldo.gil.g@urosario.edu.co](mailto:oswaldo.gil.g@urosario.edu.co) ; [ajosafat@arizona.edu](mailto:ajosafat@arizona.edu))

# Supporting information

**S4 Table. Effects on latency time during memory test. a)** LMM model to test the effects of stimulus Structure during acquisition, Intensity and the modality order employed during memory tests on latency time. Significance values following a Bonferroni’s correction. **b)** Posthoc contrasts after the LMM model contrasting the difference in the latency time between modalities of unrewarded stimuli during the memory retention test.

|  | **a.    GLMM Memory Model for Reaction time (s)** |  |  |  |  |  | |  | |  |
| --- | --- | --- | --- | --- | --- | --- | --- | --- | --- | --- |
|  | Latency time ~ *Structure during acquisition* X *Intensity* X *Stimuli during Memory* + (1 \| individual honeybee) | | | | | |  | |  | |
|  | **Contrast** | **Estimate** | **S.E** | **t value** | **p** |  | |  | |  |
|  | Intercept | 0.86 | 0.16 | 5.51 | **0.001** | *** | | | |  |
|  | (Structure/ acquisition) Visual ; Olfactory | 0.63 | 0.2 | 3.1 | **0.01** | ** | |  | |  |
|  | (Structure/ acquisition) Bimodal | 0.20 | 0.19 | 1.05 | 0.29 |  | |  | |  |
|  | (Intensity) High | 0.04 | 0.20 | 0.22 | 0.82 |  | |  | |  |
|  | (Stimuli during memory) Olfactory | 0.38 | 0.17 | 2.26 | 0.02 |  | |  | |  |
|  | (Stimuli during memory) Visual | 0.73 | 0.21 | 3.48 | **0.001** | *** | | | |  |
|  | (Structure acquisition) Visual; Olfactory X (Intensity) High | -0.68 | 0.27 | -2.54 | **0.01** | ** | |  | |  |
|  | (Structure /acquisition) Bimodal X (Intensity) High | -0.13 | 0.24 | -0.53 | 0.59 |  | |  | |  |
|  | (Structure /acquisition ) Visual; Olfactory X (stimuli during memory) Olfactory | -0.62 | 0.22 | -2.82 | **0.001** | *** | | | |  |
|  | (Structure /acquisition) Bimodal X (stimuli durung memory) Olfactory | -0.46 | 0.20 | -2.27 | **0.02** | * | |  | |  |
|  | (Structure /acquisition) Visual ; Olfactory X (stimuli during memory) Visual | -0.51 | 0.27 | -1.89 | 0.06 |  | |  | |  |
|  | (Structure /acquisition) Bimodal X (stimuli during memory) Visual | 0.02 | 0.25 | 0.08 | 0.93 |  | |  | |  |
|  | (Intensity) High X (stimuli during memory) Olfactory | -0.28 | 0.21 | -1.30 | 0.19 |  | |  | |  |
|  | (Intensity) High X (stimuli during memory) Visual | 0.08 | 0.32 | 0.24 | 0.81 |  | |  | |  |
|  | (Structure / acquisition) Visual ; Olfactory X (Intensity) High X (stimuli during memory) Olfactory | 0.61 | 0.28 | 2.15 | **0.03** | * | |  | |  |
|  | (Structure / acquisition) Bimodal X (Intensity) High X (stimuli during memory) Olfactory | 0.37 | 0.27 | 1.41 | 0.16 |  | |  | |  |
|  | (Structure /acquisition) Visual ; Olfactory X (Intensity) High X (stimuli during memory) Visual | 0.77 | 0.40 | 1.91 | 0.06 |  | |  | |  |
|  | (Structure /acquisition) Bimodal X (Intensity) High X (stimuli during memory) Visual | -0.16 | 0.39 | -0.42 | 0.67 |  | |  | |  |

| **b. Posthoc contrasts** |  |  |  |  |
| --- | --- | --- | --- | --- |
| **Low intensity** | | | | |
| Contrasts | **estimate** | **SE** | **t.ratio** | **p** |
| Bimodal - Olfactory | -0.0185 | 0.0825 | -0.224 | 0.9726 |
| Bimodal - Visual | -0.5665 | 0.0991 | -5.715 | <.0001 |
| Olfactory - Visual | -0.548 | 0.0905 | -6.057 | <.0001 |
| **High intensity** | | | | |
| Bimodal - Olfactory | -0.0689 | 0.0705 | -0.977 | 0.5912 |
| Bimodal - Visual | -0.8458 | 0.1173 | -7.209 | <.0001 |
| Olfactory - Visual | -0.7769 | 0.1104 | -7.036 | <.0001 |
|  |  |  |  |  |
